# Dementia – Prevalence, trends and regional patterns in Germany. An analysis based on routine data from the statutory health insurance

**DOI:** 10.25646/13079

**Published:** 2025-03-31

**Authors:** Alexander Rommel, Beate Gaertner, Hannelore Neuhauser, Dinara Yessimova, Helmut Schröder, Gabriela Brückner, Katrin Schüssel, Michael Porst

**Affiliations:** 1 Robert Koch Institute, Department of Epidemiology and Health Monitoring, Berlin, Germany; 2 AOK Federal Association, WIdO – AOK Research Institute, Berlin, Germany

**Keywords:** Dementia, Alzheimer’s disease, Prevalence, Time trends, Morbidity, Risk factors, Age distribution, Health claims data, Secondary data analysis, Public health

## Abstract

**Background:**

As part of the German Burden of Disease Study, population-based prevalences of important diseases are estimated. This allows regional patterns and temporal trends to be identified.

**Methods:**

The prevalence of dementia in the population was estimated cross-sectionally for the years 2017 to 2022 at the level of the Spatial Planning Regions using routine data of persons insured in the statutory health insurance AOK, adjusted for age, sex and morbidity (administrative prevalence).

**Results:**

In 2022, the prevalence of dementia in Germany was 2.8% of the population aged 40 and over. In women the prevalence was 3.3 %, in men 2.4 %. The prevalence of dementia rises sharply with age. For example, the prevalence among people aged 65 and over was 6.9 %. A slight downward trend was observed between 2017 and 2022. The age-standardised regional distribution shows a clear pattern of higher prevalence in eastern Germany and the eastern part of Bavaria.

**Conclusions:**

Measured by administrative prevalence, the public health significance of dementia remains largely stable. However, demographic change is expected to increase the number of people affected by dementia. Prevention of modifiable risk factors is therefore essential, especially in middle age.

This article is part of a series of articles with standardised analyses for the German Burden of Disease Study of the Robert Koch Institute.

## 1. Introduction

In order to support health policy decisions, the evaluation of the burden of disease in the population is of growing importance. Burden of disease indicators represent the ‘loss’ of life years at the level of population health caused by health impairments and premature death. The methods were originally developed by the Global Burden of Disease Study (GBD) [1–3].

Disease burden indicators make it possible to compare the impact of different diseases and to draw conclusions about regional differences and trends in population health over time. As part of the German Burden of Disease Study, this methodology is adapted and applied to diseases and injuries of high public health relevance [4, 5].

In order to calculate the morbidity-related burden of disease, prevalences of diseases and injuries are needed. These alone are of great value for public health research and fill existing information gaps for diseases for which comprehensive epidemiologic descriptions are rare or lacking.

**Key messages**

► In 2022, 2.8 % of people aged 40 and over in Germany had a diagnosed dementia.
► The prevalence is 3.3 % for women and 2.4 % for men.
► The prevalence of dementia rises sharply with age, with a rate of 6.9 % among those aged 65 and over.
► The regional distribution shows higher prevalences in eastern Germany.
► Over time, the prevalence decreased slightly between 2017 and 2022.

Dementia is an important cause of the burden of disease in the population and is of high public health relevance (Infobox). This article reports on the prevalence of dementia as measured by the Robert Koch Institute’s Burden of Disease Study. It is in line with the standard for reporting secondary data analyses in Germany [6].

## 2. Methods

The present analysis is based on routine data of persons insured in the statutory health insurance (SHI) system. These data are mainly generated by cost accounting between service providers (e.g. hospitals) and payers (health insurance funds) in the health care system and are only subsequently made available for research purposes (secondary data analysis). Routine SHI data are collected continuously and allow trend analyses as well as small-area analyses. The data contain the most important information for estimating the prevalence: (i) diagnoses according to the 10th revision of International Statistical Classification of Diseases and Related Health Problems (ICD-10-GM), (ii) services according to the official classification for the coding of surgeries, procedures and general medical measures (OPS) and (iii) drug prescriptions that can be categorised using the pharmaceutical central number (PZN) of the classification according to the Anatomical Therapeutic Chemical (ATC) system [13].

The underlying methodology for calculating prevalences based on routine SHI data consists of three steps: first, the definition of the prevalence concept in the insured population (see 2.1), second, the development of the case definition for identifying diseased persons (case selection criteria, see 2.2), and third, an age-, sex- and morbidity-adjusted extrapolation of the prevalence rates to the whole population using regression analysis. This allows statements to be made for all residents in the regions of Germany (see 2.3).

**Infobox Dementia**

Dementia is characterised by a progressive and irreversible deterioration of brain structures associated with a decline in cognitive abilities such as memory, language, attention and concentration, as well as changes in personality, emotions and social skills [7]. The disease develops gradually over several years or decades. It can have a variety of causes and can vary in severity and progression. People with dementia find that their ability to carry out everyday activities and maintain an independent lifestyle becomes increasingly limited, to the point where they need support and care. There are different types of dementia. Alzheimer’s disease accounts for about two-thirds of all cases of dementia [8]. The second most common form is vascular dementia, caused in part by repeated small strokes [9]. Medications and non-medical treatments (e.g. memory training) can help slow the progression of the disease, but it cannot be cured.

The prevalence of most types of dementia does not start to increase significantly until around the age of 65. However, dementia can occur at a younger age, such as frontotemporal dementia, which mainly affects the frontal and temporal lobes of the brain and is associated with behavioural problems [10]. The modifiable risk factors for dementia include social (low level of education, social isolation) and environmental (air pollution) conditions, behavioural (smoking, physical inactivity, alcohol consumption) and metabolic (high blood pressure, elevated LDL cholesterol, obesity) risks and certain diseases (depression, diabetes mellitus, hearing and vision loss, head injury). According to the Lancet Commission on Dementia, about half of all dementia cases could currently be delayed or prevented by avoiding modifiable risk factors [11].

Dementia is associated with complex care needs and high levels of dependency and morbidity in the later stages. Dementia therefore requires a range of services, both within and outside the health sector, such as primary health care, specialised health care, community-based services, rehabilitation, long-term care and palliative care. The World Health Organization (WHO) recommends that primary health care be integrated into multidisciplinary and modular models of care, taking into account the needs of both the person with dementia and their carers [12].

### 2.1 Insured population and prevalence concept for measuring 1-year prevalence of dementia

Pseudonymised routine data from around 27 million AOK insurance policyholders from the years 2017 to 2022 is analysed using a cross-sectional approach to identify people affected by a disease [14, 15]. Prevalence is defined as the proportion of persons affected by a disease during the analysis period out of the total number of people included in the study. In analyses using routine SHI data, it should be considered that the underlying population of insured persons is an open, dynamic cohort with inflows and outflows due to natural population movements (births, deaths) or changes in an individual’s insurance history (e.g. change of health insurance company). Therefore, all calculations are not based on individuals but on observed insurance periods in days [16]. In this way, insurance periods of new-borns or deceased persons, as well as those of persons who change insurance, can be considered on a pro rata basis. The period of insurance and the regional allocation of the insured is determined on a quarterly basis. Finally, the population of insured persons, and thus the denominator of the prevalence estimate, is obtained as the total number of observed quarterly insurance periods for the respective reference year [16].

### 2.2 Case definition for dementia

A case definition for the inclusion of persons with prevalent dementia has been developed in collaboration with renowned internal and external experts. The period analysed always refers to 12 months. Inclusion criteria are based on ICD-10-GM coded diagnoses (Table 1). Further information such as prescriptions of medication was not considered.

As the diagnoses included are highly age-associated, diagnoses of dementia in persons under the age of 40 were not included. Accordingly, diagnoses of dementia for all persons in the population of insured persons aged 40 and over were considered when determining the number of persons affected (numerator). The criteria were applied to all persons in the insured population in each quarter of the reference year, looking back three quarters from the reference quarter to determine 1-year prevalence. Finally, to determine the number of persons affected by a disease and thus the numerator of the prevalence calculation, the observed person-time of the cases in each quarter of a calendar year was summed up.

### 2.3 Statistical methods

Since the group of policyholders of a health insurance fund is not a random sample of the general population and is therefore not representative of the population [15, 17–20], the specific prevalence estimates for each health insurance fund must be extrapolated to the whole population. Due to the regionally different distribution of the population in each health insurance fund, this extrapolation is done by region [21]. In this regression analysis, regionally available statistics on the frequency of inpatient diagnoses and on the demographic structure of the population on the level of the 400 German districts are used as auxiliary information [22, 23]. In this way, in addition to demographic differences, morbidity differences between health insurance funds and the German population can be corrected (morbidity-adjusted) and differentiated by small areas. The method was developed and its plausibility tested using type 2 diabetes as an example [21]. It has been adapted for dementia to estimate the prevalence for the whole population of Germany at the level of the 96 Spatial Planning Regions for dementia for each reference year.

When extrapolating prevalences, individual age groups are combined into larger age groups for model stability, so that a prevalence is not always available for each 5-year age group. To allow stratification at this level of detail, a special procedure is used to model missing age-specific prevalence rates. For this purpose, the sex-specific prevalence patterns of the AOK population along the 5-year age groups (raw data) are transferred to the (pooled) age groups of the extrapolation. The extrapolated total prevalence in the combined age group serves as the target value for the modelling. The statistical uncertainty is derived from the variance of the morbidity-adjusted total prevalence. In addition, the results are age-standardised using the European Standard Population 2013 [24] for the presentation of maps and time trends.

## 3. Results

**Figure 1:**
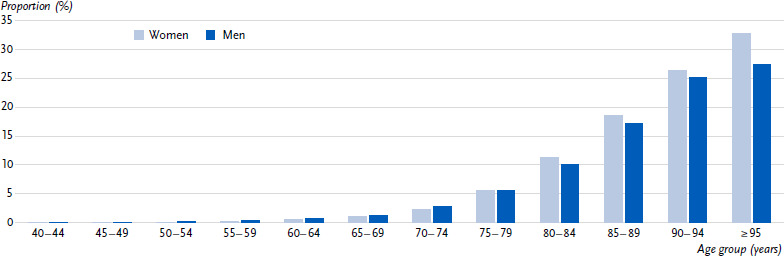
Prevalence of dementia by age and sex (population aged 40 and over in *%).* Source: Burden of Disease Study for Germany (AOK routine data 2022, age-, sex- and morbidity-adjusted and extrapolated to the German population)

In 2022, 2.8 % of the population aged 40 and over in Germany was affected by dementia (administrative prevalence). This corresponds to nearly 1.4 million people. The prevalence is 3.3 % for women and 2.4 % for men. The prevalence of dementia rises sharply with age. Among people aged 65 and over, the prevalence is 6.9 %. Among those aged 95 and over, 32.7 % of women and 27.4 % of men have dementia. Gender differences in terms of a higher prevalence of dementia in women exist particularly among the very old (Figure 1, Annex Table 1).

**Figure 2:**
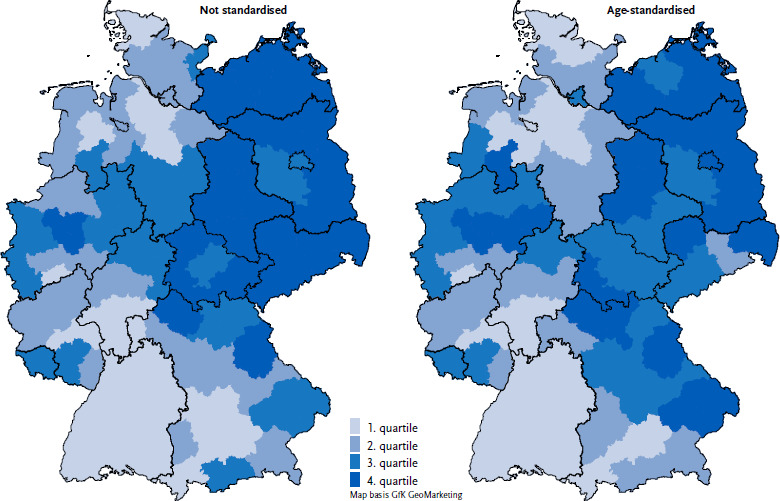
Prevalence of dementia on the level of the Spatial Planning Regions (population aged 40 and over in *%*, quartiles).. Source: Burden of Disease Study for Germany (AOK routine data 2022, age-, sex- and morbidity-adjusted and extrapolated to the German population)

Even after age standardisation, the regional distribution shows a clear pattern of higher prevalence of dementia in some regions in western Germany and Bavaria, and particularly in the eastern federal states. The difference is therefore not primarily due to the fact that the population in these regions is older on average (Figure 2).

**Figure 3:**
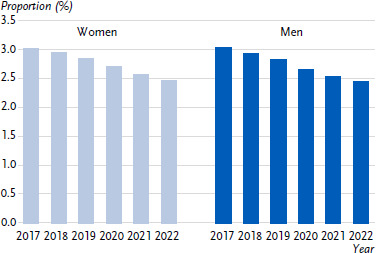
Prevalence of dementia over time (population aged 40 and over in %, standardised by age). Source: Burden of Disease Study for Germany (AOK routine data 2017 – 2022, adjusted for age, sex and morbidity and extrapolated to the German population)

Over time, the prevalence of dementia in Germany declined by nearly 0.6 percentage points between 2017 and 2022 (Figure 3, Annex Table 2). The largest decline was among people aged 95 and over, by around 6 percentage points, from 37.9 % to 31.5 %.

## 4. Discussion

In Germany, an estimated 2.8 % of the population aged 40 and over was diagnosed with dementia in 2022. Among those aged 65 and over, the prevalence is 6.9 %. Among the very old, the prevalence is significantly higher among women than among men. Dementia is a group of diseases that are strongly associated with age and increase sharply in older age groups. It is very rare in younger people. The regional distribution shows higher age-standardised prevalence in eastern Germany, but also in parts of Bavaria, the Ruhr area and Saarland. Over time, the prevalence of dementia is decreased between 2017 and 2022.

The AOK Health Atlas uses a comparatively less strict definition and reports a prevalence that is about 0.3 percentage points higher. In addition, many studies estimate the absolute number of people with dementia in Germany at between 1.6 and 1.8 million, which is higher than the 1.4 million reported here [7, 25–27]. This may be partly due to the fact that only cases with a confirmed diagnosis and a follow-up diagnosis within a one-year period were included in the present study. However, it should be noted that many of the higher figures are also subject to uncertainty, as they are partly based on international estimates that are not directly derived from German data [25, 27–29]. The results also show a lower prevalence of dementia in Germany than estimates from the international GBD study. The GBD study reports a population prevalence of 2.45 % for Germany, which is almost as high as the prevalence reported here for people aged 40 and over. The AOK Health Atlas calculates a prevalence of only 1.7 % for the total population [30]. Furthermore, the prevalence for Germany in the GBD study is significantly higher than for the USA (1.5 %), the United Kingdom (1.4 %) or Austria (1.7 %). In addition, the GBD generally estimates a further increase in prevalence over time [31]. This trend is not consistent with the present findings and other studies, which predominantly assume a decreasing relative prevalence of dementia [30, 32]. As the GBD study is based on modelling a wide range of international data sources, these discrepancies are difficult to explain.

Furthermore, the results are in many respects consistent with previous evidence [7, 26, 33]. Due to the higher prevalence in women, especially in older age groups, and the longer life expectancy of women, about two thirds of people with dementia are female [25]. The reasons for this are not fully understood. In addition to medical factors, the lower education of women in older birth cohorts has been suggested [34, 35]. The declining prevalence over time confirms earlier findings from analyses of German routine data [26, 30] and from international cohort studies, most of which also report declining prevalences for dementia [11, 32, 36, 37]. The reasons for this are thought to be increasing levels of education and a reduction in cardiovascular risk factors such as smoking, as well as improved treatment of cardiovascular diseases [11, 26, 32, 36]. However, also in Germany the decline in prevalence will be more than offset by the demographic ageing of the population. As a result, the absolute number of people with dementia is expected to increase significantly over the next few decades [25], but may begin to decline from around 2050 under the assumption of preventive effects [7].

The regional pattern in the prevalence of dementia is also striking. Especially the higher prevalences in the eastern German regions, Saarland and some regions of North Rhine-Westphalia, reflect the regionally uneven distribution of social deprivation in Germany. Social deprivation is determined, among other things, by income and employment and is associated, for example, with risk factors for cardiovascular dementia [38, 39]. In particular, dementia risk factors that were regionally unevenly distributed 30 years ago and were more common in eastern Germany (e.g. diabetes, hypertension, obesity, hazardous alcohol consumption), may play an important role [39, 40]. As the administrative prevalence of some of these risk factors (type 2 diabetes, hypertension, obesity) is still significantly higher in eastern Germany as well as in some regions of western Germany and north-eastern Bavaria [30], these regional differences may persist in the long term [30]. Other analyses based on routine data from the statutory health insurance system show a similar pattern [30, 41], whereas studies using the projection of age-specific prevalences for Germany onto the regional population structure do not show higher prevalence rates in Bavaria [27]. This suggests that the prevalence of dementia in some regions of Bavaria is higher than would be expected from the age structure of the regions. As explained above, this may be due to the regional distribution of risk factors for dementia. On the other hand, it is also conceivable that the results based on SHI data are influenced by care structures such as the regional density of physicians. For example, in the eastern part of Bavaria there is no above-average concentration of neurology practices, but a relatively high density of general practitioners [42]. Future studies should investigate the extent to which this is associated with a higher diagnosed prevalence.

The present analysis relies on routine SHI data. One advantage of such data is that some of the typical sources of error associated with primary data collection, such as surveys, are excluded. These include bias due to recall bias, non-response or lower participation of hard-to-reach groups [43]. One limitation that needs to be considered is that SHI routine data mainly contain information relevant for accounting (see 2.). Non-utilisation of health services, lack of documentation of diagnoses and financial incentives to optimise accounting can lead to misclassification and bias in the data [43, 44]. Non-utilisation is of little relevance for many conditions if they are so severe, such as strokes, that they usually lead to medical contact or hospitalisation. However, misclassification (over- or underestimation) of diseased persons can occur if diagnoses are coded incorrectly or not at all.

In order to minimise misclassifications in routine data, disease-specific case definitions were developed for each disease, which, in addition to diagnoses, use further information on surgeries, drug prescriptions or outpatient claim codes for plausibility checks [16, 44, 45]. For example, cases with a diagnosis of dementia as a secondary inpatient diagnosis or as a definite outpatient diagnosis were internally validated using the M2Q criterion (diagnosis in at least two quarters of the analysis period). Nevertheless, it can be assumed that measuring the prevalence of dementia in routine data is likely to underestimate the number of cases of dementia, as the disease is often diagnosed some time after the onset of symptoms. For example, there is evidence that the diagnosis of dementia is often delayed because of a lack of therapeutic options [46]. In summary, the present estimate is somewhat more conservative than those from studies not based on claims data.

Other limitations of the results are related to the statistical methods used for extrapolation and for modelling the age distribution in the 5-year groups. The extrapolation method uses the diagnoses of all hospital admissions in Germany to adjust for differences in morbidity between insurance funds and the population, and has been developed and validated for type 2 diabetes [21]. It is thus assumed that the estimated prevalences no longer reflect the insurance fund specific morbidity, but that of the population. To model age distribution, it was assumed that the age progression of dementia among AOK insured persons could be applied to the combined age groups from the extrapolation results. The overall prevalence remains unaffected by this procedure. To assess the plausibility of extrapolation and age modelling, the results were compared with published values for the prevalence of dementia from Germany, with good agreement of the estimates by age and sex [7, 26].

Burden of disease studies place high demands on the data to be used. Among other things, they require the most accurate information possible on the frequency of disease by age, sex and region. For many diseases, routine data from the SHI system are the preferred option for estimating and presenting prevalence at the small area level. Thus, burden of disease studies, especially when conducted regularly, provide important basic epidemiological information and fill information gaps.

Although the age-standardised prevalence of diagnosed dementia is currently declining, these diseases remain a major public health and care challenge. Forecasts for the coming decades predict that the absolute number of people affected will continue to increase due to demographic ageing [7]. Prevention of dementia (especially in middle age) is important because of the loss of independence and quality of life for people with dementia and the resulting burden on their friends and relatives [11, 25]. This includes preventing behavioural risk factors and promoting protective factors, such as good social integration in old age. In this way, the increase in the number of people with dementia could be counteracted despite demographic ageing [7, 25]. It is therefore all the more important to fully implement and maintain the National Dementia Strategy (https://www.nationale-demenzstrategie.de/english), to monitor adult cognitive function and the prevalence of dementia risk factors at population level, and to provide population-based prevention and care services for people with dementia and their friends and relatives to enable them to live as independently as possible with dementia.

## Figures and Tables

**Table 1:** Selection criteria for defining the prevalence of dementia with AOK routine data

Health care sector	Inpatient care^1^: main diagnoses	Other diagnoses		
		Inpatient care^1^: secondary diagnoses	Specialised ambulatory care^2^	Ambulatory care in medical practices^3^
**Inclusion criteria**				
**Criterion**	At least one diagnosis in the analysis period	Diagnosis in at least two quarters in the analysis period^4^		
**Codes**	ICD-10-GM: F00 Dementia in Alzheimer disease F01 Vascular dementia F02 Dementia in other diseases classified elsewhere F03 Unspecified dementia G30 Alzheimer disease G31.0 Circumscribed brain atrophy G31.82 Lewy body(ies) (dementia) (disease)			

^1^ Inpatient cases (§ 301 para. 1 SGB V): Main and secondary diagnoses of the complete inpatient and day patient cases (discharge diagnoses)

^2^ Cases of specialised ambulatory care (§§ 115b, 116b, 117 para. 1 to 3, 118, 119, 119c, 120, 140a SGB V) (mainly ambulatory care in hospitals)

^3^ Cases of ambulatory care in medical practices paid under the scheme of statutory health insurance (§ 295 para. 2 SGB V)

^4^ So called M2Q-criterion

ICD-10-GM = International Statistical Classification of Diseases and Related Health Problems, 10th Revision, German Modification, SGB=Social Security Act

**Annex Table 1:** Prevalence of dementia by age and sex (population aged 40 and over in %). Source: Burden of Disease Study for Germany (AOK routine data 2022, adjusted for age, sex and morbidity and extrapolated to the German population)

Age group (years)	Women	Men	Total
	%	%	%
40–44	0.04	0.05	0.05
45–49	0.09	0.07	0.08
50–54	0.12	0.21	0.17
55–59	0.29	0.37	0.33
60–64	0.61	0.67	0.64
65–69	1.05	1.38	1.21
70–74	2.31	2.87	2.57
75–79	5.58	5.57	5.57
80–84	11.26	10.11	10.78
85–89	18.55	17.12	18.01
90–94	26.30	25.07	25.92
≥95	32.72	27.36	31.53

**Annex Table 2:** Prevalence of dementia over time (population aged 40 and over in %, crude and age-standardised). Source: Burden of Disease Study for Germany (AOK routine data 2017-2022, age-, sex- and morbidity-adjusted and extrapolated to the German population)

Year	Women (not standardised)	Men (not standardised)	Total (not standardised)	Women (age-standardised)	Men (age-standardised)	Total (age-standardised)
	%	%	%	%	%	%
2017	3.81	2.69	3.27	3.02	3.03	3.05
2018	3.76	2.66	3.24	2.95	2.93	2.97
2019	3.70	2.64	3.19	2.84	2.83	2.86
2020	3.56	2.52	3.06	2.70	2.65	2.70
2021	3.42	2.44	2.95	2.57	2.53	2.57
2022	3.26	2.37	2.84	2.46	2.44	2.47
